# Supplementary material for: Factors associated with shisha smoking: Results from a cross-sectional telephone-based survey among the general population adults in Nigeria
Source: Tob Prev Cessat. 2025 Jan 17;11:10.18332/tpc/194632. doi: 10.18332/tpc/194632 (PMC11740602; doi:10.18332/tpc/194632)
Supplement: Supplementary file 1 [file TPC-11-05-s1.pdf]

Supplementary figure: Number of participants by current shisha smoking status and geopolitical zone

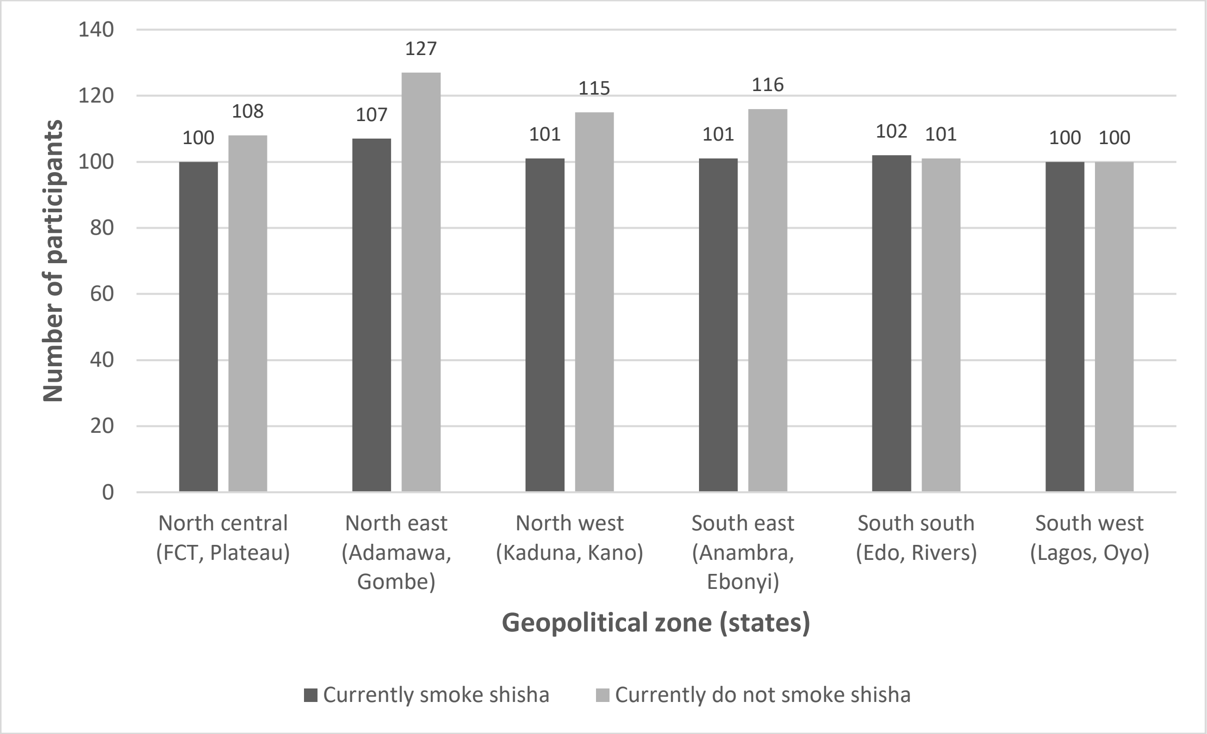

Supplementary table: Factors associated with shisha smoking by sex

	Males		Females	
**Variable**	**[a]cOR (95% CI)**	**[b]aOR (95% CI)**	**[a]cOR (95% CI)**	**[b]aOR (95% CI)**
**Age**	0.90 (0.88 to 0.93)	**0.92 (0.90 to 0.95)**	0.89 (0.85 to 0.93)	**0.90 (0.82 to 0.98)**
**Urban/rural**				
Rural	1	1	1	1
Urban	1.64 (0.89 to 3.02)	1.17 (0.59 to 2.31)	1.70 (0.86 to 3.38)	2.30 (0.39 to 13.68)
**Level of education**				
No education	1	1	1	1
Primary	0.83 (0.18 to 3.89)	0.32 (0.05 to 2.13)	2.53 (0.24 to 26.78)	0.93 (0.10 to 8.91)
≥ Secondary	9.04 (2.64 to 30.87)	0.46 (0.20 to 1.06)	10.86 (1.43 to 82.73)	0.37 (0.02 to 6.71)
**Religion**				
Christianity	1	1	1	1
Islam	0.68 (0.50 to 0.94)	0.91 (0.65 to 1.27)	0.32 (0.16 to 0.65)	0.85 (0.35 to 2.04)
None	0.75 (0.28 to 2.00)	0.75 (0.18 to 3.22)	1.70 (0.12 to 24.17)	0.68 (0.06 to 7.41)
**Wealth quintile**				
Poorest	1	1	1	1
Poorer	0.49 (0.36 to 0.68)	0.83 (0.44 to 1.60)	0.62 (0.39 to 0.98)	0.78 (0.26 to 2.39)
Middle	0.48 (0.28 to 0.81)	0.68 (0.34 to 1.37)	0.20 (0.03 to 1.52)	0.37 (0.11 to 1.22)
Richer	0.25 (0.16 to 0.39)	**0.39 (0.17 to 0.88)**	0.12 (0.05 to 0.33)	0.43 (0.07 to 2.60)
Richest	0.10 (0.06 to 0.17)	0.35 (0.11 to 1.12)	0.17 (0.08 to 0.38)	1.85 (0.12 to 27.69)

**Employment status**				
Unemployed	1	1	1	1
Employed	1.63 (0.54 to 4.89)	**2.53 (1.09 to 5.89)**	2.02 (0.94 to 4.34)	0.79 (0.09 to 6.71)
Student	3.73 (1.22 to 11.38)	**3.85 (1.43 to 10.41)**	5.13 (1.76 to 14.98)	0.84 (0.18 to 4.06)
Apprentice	1.38 (0.75 to 2.54)	2.77 (0.77 to 9.96)	1.47 (0.86 to 2.50)	0.09 (0.00 to 4.27)
**At least one family member smokes shisha**				
No	1	1	1	1
Yes	9.40 (4.02 to 22.01)	**2.23 (1.16 to 4.29)**	13.58 (6.31 to 29.25)	**4.91 (0.88 to 27.26)**
**Number of shisha smokers among closet friends**				
0	1	1	1	1
1	12.85 (4.15 to 39.79)	**10.81 (4.17 to 28.00)**	176.40 (46.68 to 666.52)	**289.38 (34.94 to 2396.71)**
2+	114.98 (34.74 to 380.55)	**64.17 (21.68 to 189.97)**	327.47 (42.01 to 2552.79)	**226.58 (20.12 to 2552.09)**
Don't know	2.45 (0.28 to 21.39)	3.59 (0.27 to 47.66)	1.05 (0.04 to 27. 35)	1.98 (0.09 to 44.70)
**Currently smoke cigarettes**				
No	1	1	N/A	N/A
Yes	7.20 (3.16 to 16.42)	**4.62 (2.24 to 9.51)**	N/A	N/A
**Currently consumes alcohol**				
No	1	1	1	1
Yes	4.49 (3.27 to 6.16)	**2.82 (1.47 to 5.40)**	16.73 (7.84 to 35.71)	**8.88 (2.93 to 26.95)**

**PSS-4**	0.83 (0.77 to 0.89)	**0.85 (0.72 to 0.99)**	0.91 (0.83 to 1.01)	1.10 (0.93 to 1.30)
**PHQ-2**				
Negative	1	1	1	1
Positive	0.66 (0.47 to 0.92)	0.85 (0.46 to 1.56)	0.68 (0.46 to 1.00)	**0.36 (0.17 to 0.81)**
**GAD-2**				
Negative	1	1	1	1
Positive	0.54 (0.42 to 0.70)	0.61 (0.32 to 1.14)	0.66 (0.44 to 0.99)	0.42 (0.15 to 1.15)

[a]crude/unadjusted odds ratios

[b]Adjusted odds ratios

© 2025 Mdege N.D. et al.
